# Supplementary material for: A Transcriptome and Methylome Study Comparing Tissues of Early and Late Onset Colorectal Carcinoma
Source: Int J Mol Sci. 2022 Nov 17;23(22):14261. doi: 10.3390/ijms232214261 (PMC9697435; doi:10.3390/ijms232214261)
Supplement: Supplementary file 1 [file ijms-23-14261-s001.zip › Supplemental Figures CRC.pdf]

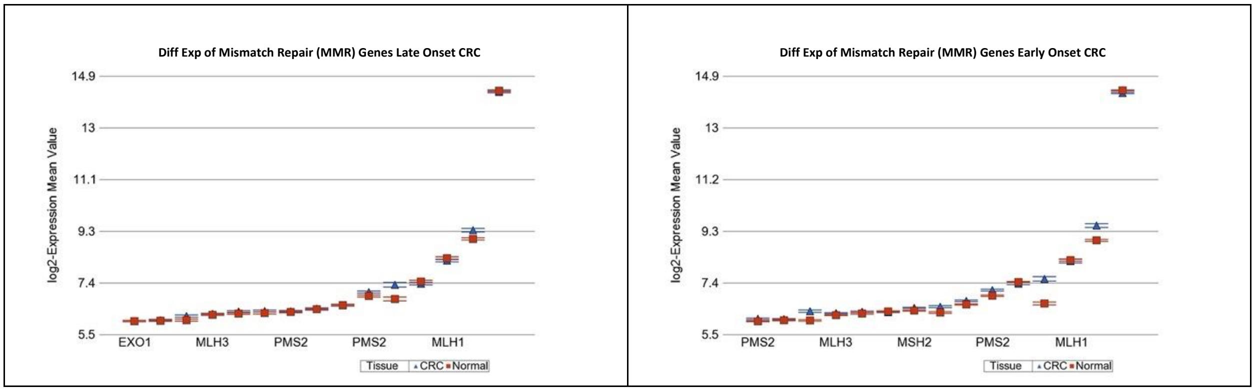

Figure S1: Differential expression of MMR genes in LOCRC and EOCRC

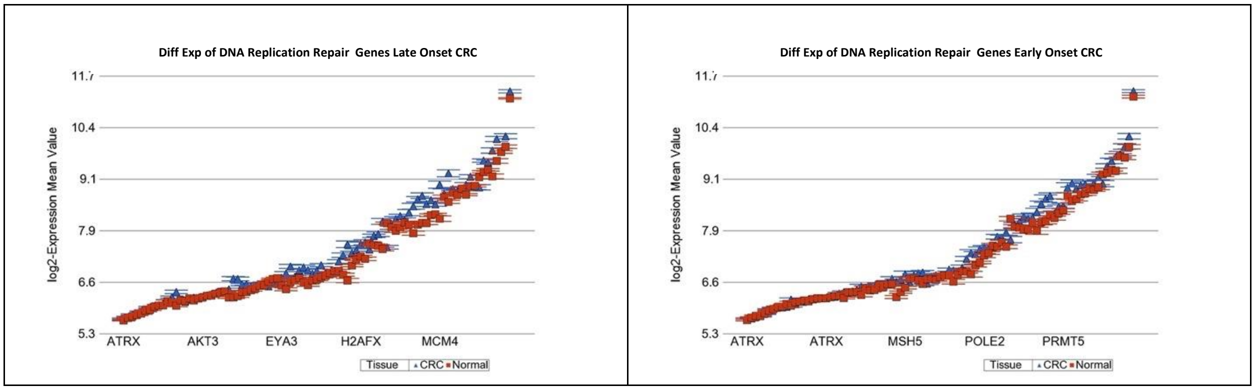

Figure S2: Differential expression of DNA replication repair genes in LOCRC and EOCRC

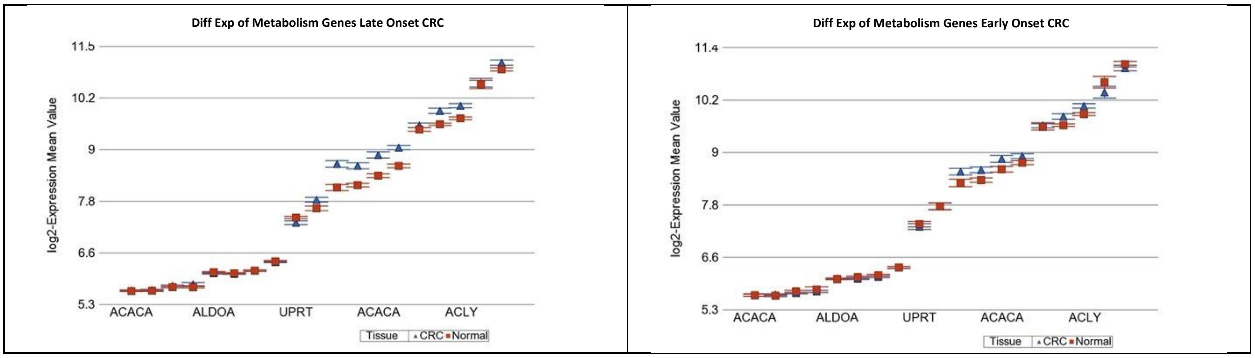

Figure S3: Differential expression of metabolism genes in LOCRC and EOCRC

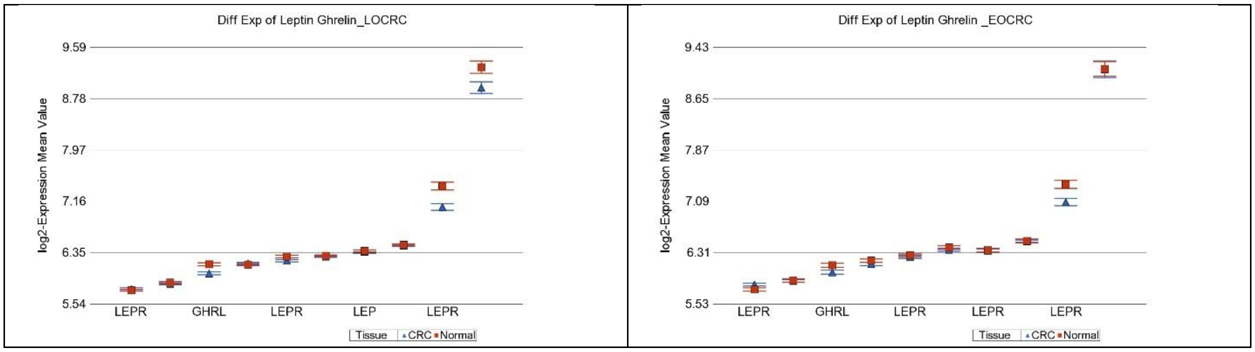

Figure S4: Differential expression of obesity related genes in LOCRC and EOCRC

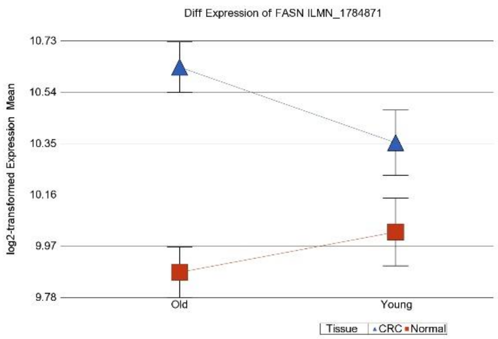

Figure S5: Differential expression of FASN gene in LOCRC and EOCRC

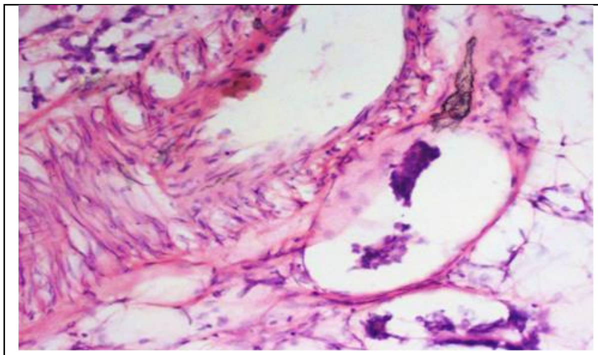

Figure S6A

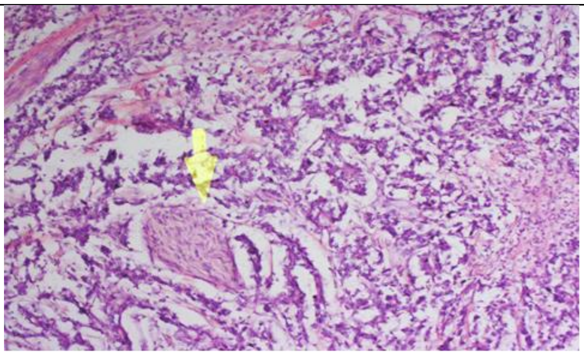

Figure S6B

Figure S6: Photo micrograph HE stain at 200x magnification showing LVI (shown in S6A) and PNI (shown in S6B)
